# Use of Formalin-Fixed Paraffin-Embedded Samples for Gene Expression Studies in Breast Cancer Patients

**DOI:** 10.1371/journal.pone.0123194

**Published:** 2015-04-06

**Authors:** Valeria Musella, Maurizio Callari, Eleonora Di Buduo, Manuela Scuro, Matteo Dugo, Patrizia Miodini, Giampaolo Bianchini, Biagio Paolini, Luca Gianni, Maria Grazia Daidone, Vera Cappelletti

**Affiliations:** 1 Department of Experimental Oncology and Molecular Medicine, Fondazione IRCCS Istituto Nazionale dei Tumori, Milan, Italy; 2 Department of Pathology, Fondazione IRCCS Istituto Nazionale dei Tumori, Milan, Italy; 3 Department of Medical Oncology, Ospedale San Raffaele, Milan, Italy; University of Torino, ITALY

## Abstract

To obtain gene expression profiles from samples collected in clinical trials, we conducted a pilot study to assess feasibility and estimate sample attrition rates when profiling formalin-fixed, paraffin-embedded specimens. Ten matched fresh-frozen and fixed breast cancer samples were profiled using the Illumina HT-12 and Ref-8 chips, respectively. The profiles obtained with Ref 8, were neither technically nor biologically reliable since they failed to yield the expected separation between estrogen receptor positive and negative samples. With the use of Affymetrix HG-U133 2.0 Plus chips on fixed samples and a quantitative polymerase chain reaction -based sample pre-assessment step, results were satisfactory in terms of biological reliability, despite the low number of present calls (M = 21%±5). Compared with the Illumina DASL WG platform, Affymetrix data showed a wider interquartile range (1.32 vs 0.57, *P*<2.2 E-16,) and larger fold changes. The Affymetrix chips were used to run a pilot study on 60 fixed breast cancers. By including in the workflow the sample pre-assessment steps, 96% of the samples predicted to give good results (44/46), were in fact rated as satisfactory from the point of view of technical and biological meaningfulness. Our gene expression profiles showed strong agreement with immunohistochemistry data, were able to reproduce breast cancer molecular subtypes, and allowed the validation of an estrogen receptor status classifier derived in frozen samples. The approach is therefore suitable to profile formalin-fixed paraffin-embedded samples collected in clinical trials, provided that quality controls are run both before (sample pre-assessment) and after hybridization on the array.

## Introduction

The impact of gene expression profiling on the development of personalized care in recent years has been enormous. Gene expression profiling by microarray technology now allows the classification of breast cancers into distinct molecular subtypes and the prediction of prognosis and response to treatment [1–4].

However, the successful application of the microarray tool to measure gene expression in the clinical setting took a long time not only due to specific technical issues in hybridization techniques, but also due to the necessity to rely on fresh frozen (FF) tissue samples and on organized tissue banks with well-annotated samples linked to clinico-pathologic and follow-up data. The possibility to bypass the requirement of fresh tissue and to work directly on formalin-fixed paraffin-embedded (FFPE) samples would allow access to a much larger number of tissue samples stored in almost every pathology department worldwide, quickly obtaining useful data from samples collected in clinical trials [5].

Extraction of RNA from FFPE samples is no longer impossible and many commercial kits are available to process FFPE tissue sections [6–11]. However, extraction yields are low and, more importantly, the RNA obtained is heavily degraded and chemically modified [12]. Furthermore, FFPE samples, suffer from pre-analytical variability as frequently also happens with frozen samples [13,14] and the heavy work-load in pathology departments often hinders a strict standardization of fixation procedures. Although delays in fixation and the absence of standardization in fixation procedures are difficult to resolve, even up to 3 decades-old adequately fixed samples can be processed for RNA extraction and the extracted RNA can be chemically de-modified and in most cases is suitable for amplification with exponential or linear procedures [15–17].

A number of studies have shown the feasibility of obtaining whole genome (WG) expression profiles from FFPE samples [18–22], but usually such samples have been processed with quantitative polymerase chain reaction (qPCR)–based techniques and give information on a limited number of genes [23]. Indeed, the preconception that biologically meaningful information is difficult to derive from poor quality fragmented RNA is still preventing the wide use of FFPE samples. However, in the recent years, the number of published clinical studies based on the use of FFPE samples, has increased especially due to the use of techniques specifically developed for degraded RNA, such as the cDNA-mediated annealing selection extension ligation (DASL) assay developed by Bibikova et al. [24], which has yielded clinically relevant results [25–31].

An additional approach which has been proposed to profile FFPE samples is the Almac Xcel Array (Almac, Durham, NC). The Almac assay was designed to create disease specific high density arrays optimized for use with FFPE samples and contains about 70% of the probes present in the Affymetrix HG-U133 Plus 2.0 arrays (Santa Clara, CA). The use of such arrays is not very diffused in clinical studies although in a 2010 paper, the Almac DSA colon cancer arrays were shown to outperform the Affymetrix chips [32].

Nonetheless, it has been demonstrated that biologically relevant information can also be acquired from FFPE samples processed with Affymetrix HG-U133 microarrays [16,33]. Such a possibility is very important, as it allows a direct comparison with large databases of clinical studies, most of which were profiled with the same platform. As emphasized by Linton et al [33], fixation and RNA degradation do not necessarily hinder gene profiling. Rather it is the variability associated with routine processing of such samples that can affect reliability of gene expression data, thereby creating a sort of batch-effect among samples, which can seriously impact the possibility to generate valid prognostic or predictive signatures from FFPE samples.

Such a problem can only be solved by running careful quality controls before (sample pre-assessment) and after hybridization on the array and by carrying out pilot studies on samples comparable to those derived from the clinical trial where gene expression profiling is going to be run. Only in this way will it be possible to obtain robust technical protocols to assess biomarkers on routinely archived material, in this way providing access to patient series included in clinical trials with accurate and mature follow-ups, and achieving level-I evidence [34].

With the final aim of obtaining gene expression profiles (GEPs) from FFPE samples collected within clinical trials, we have developed a technical protocol that gives robust and biologically reliable GEPs with the Affymetrix HG-U133 Plus 2.0 chips from 10 to20 years old breast cancer FFPE samples. The reliability and failure rates of the protocol were tested on a pilot study of 60 primary breast cancers. A critical analysis of results obtained from the pilot study is reported herein, along with our preliminary experiments comparing the performance of the Affymetrix HG-U133 Plus 2.0 chips with the Illumina WG DASL approach and the Illumina Ref-8 chips.

## Materials and Methods

### Patient sample

All patient specimens used in the study were obtained from leftover material available after diagnostic procedures in consented patients and are listed below.

- Ten samples from primary breast cancer patients undergoing surgery between 1997–2001 at the Fondazione IRCCS Istituto Nazionale Tumori of Milan (INTM) were selected based on the availability of matched samples of FFPE and FF tissue. Eight samples were estrogen receptor (ER)-positive (ER+) and two were ER-negative (ER-) according to the radioligand assay (RLA).
- Twelve FFPE samples from breast cancer patients undergoing surgery in 1992 were selected in order to have 6 ER+ and 6 ER- samples. All samples were from primary tumors and ER status was determined by RLA as previously described [35] on FF tissue at the time of surgery. Immunohistochemical (IHC) determination of ER was also carried out according to international guidelines [36], and only samples with concordant ER status by the two techniques (about 80%) were included.
- Sixty FFPE samples from consecutive patients with G_2_-G_3_ breast cancers who underwent radical mastectomy at the INTM, receiving adjuvant chemotherapy between 1998 and 2002 were selected to be comparable to samples deriving from clinical trials with long follow-up times. Sixty-five percent of patients were ER+.

### Ethics statements

The patients signed an informed consent and this study was approved by the Institutional Review Board and Independent Ethics Committee of INTM.

### RNA extraction

#### Frozen samples

Tissue (50–100 mg) was pulverized using a Mikrodismembrator (Braun Biotech International, Germany). Total RNA was extracted with the Trizol reagent (Invitrogen, Carlsbad, CA) according to the manufacturer’s instructions with an additional DNase digestion using the RNeasy kit (Qiagen, Valencia, CA).

RNA concentrations were spectrophotometrically determined with the NanoDrop ND-100 Spectrophotometer (NanoDrop Technologies, Wilmington, DE) whereas RNA quality was assessed with the Agilent 2100 Bioanalyzer (Agilent Technologies, Palo Alto, CA) using the RNA 6000 NanoLabkit (Agilent Technologies). The RNA integrity number (RIN) was determined using the software provided by the manufacturer.

#### FFPE samples

RNA from FFPE material was extracted using the miRNeasy FFPE kit (Qiagen, Valencia, CA). A 4 μm pre-cut section was stained with haematoxylin and eosin and reviewed by a pathologist. Only blocks with > 40% tumor cells were used as assessed on adjacent sections.

From FFPE blocks, 20-μm sections were cut. Extraction was done essentially according to the manufacturer’s instructions, except for Proteinase K digestion, which was run in a thermal block for 2 h at 55 C° under shaking followed by 15 min at 80°C. RNA concentrations were spectrophotometrically determined with the NanoDrop ND-100 Spectrophotometer (NanoDrop Technologies, Wilmington, DE).

### RNA amplification and purification

FFPE RNA (100 ng) in 5 μL of RNA-free water was used for retrotranscription into double-stranded cDNA and linearly amplified using the WT-Ovation FFPE RNA Amplification System v2 (NuGEN Technologies; San Carlos, CA) according to the manufacturer’s recommendations. To purify the cDNA the QIAquick PCR purification Kit (Qiagen, Valencia, CA) was used, with some modifications introduced by the NuGEN protocol.

To 160 μL of amplified cDNA, 800 μL of PB buffer were added. Thereafter the columns were washed 2 times, each with 700 μL of ethanol 80%.

### Array experiments

Array experiments with the Illumina platforms where run by the Functional Genomics Unit/Service from the INTM, whereas hybridizations on Affymetrix chips were run by an external Service Provider (DNAVision, Charleroi, Belgium).

#### Illumina chip: frozen samples

RNA FF samples were processed for microarray hybridization using the Illumina HumanHT-12 v3 chips (48,803 probes, 15 beads/probe); 800 ng of total RNA was reverse transcribed, labeled with biotin and amplified overnight using the Illumina RNA TotalPrep Amplification kit (Ambion, Austin, TX) according to the manufacturer’s protocol. One μg of the biotinylated cRNA sample was mixed with the Hyb E1 hybridization buffer containing 37.5% (w/w) formamide and then hybridized at 58°C overnight to the HumanHT-12 chip (Illumina).

Array chips were washed with manufacturer’s E1BC solution, stained with 1 μg/mL Cy3-streptavidine (Amersham Biosciences, Buckinghamshire, UK), and eventually scanned with the Illumina BeadArray Reader.

#### Illumina chip: FFPE samples

After purification the amplified single-stranded cDNA obtained from WT-Ovation FFPE RNA Amplification System v2, was labeled using a NuGen protocol for the Illumina chips. Five μL of UNG Buffer [10 mM Phosphate buffer (Sigma-ALDRICH, Inc, St.Louis, MI) and 4 mM MgCl_2_ (USB, Corporation, Cleveland, OH) and 5 μL of UNG enzyme (Epicentre Biotechnologies, Madison WI), were added to 25 μL of total volume containing 5 μg of cDNA. The solution was incubated at 50°C for 30 min in a thermal cycler. Following the incubation, 5 μL of labeling buffer [0.952 M acetic acid (Sigma-ALDRICH Inc St.Louis, MI) 28 mM MgCl_2_ (USB), 5 μL of ARP solution [11.2 mg/mL ARP (N-aminooxyacetyl)-N’-(D-biotinoyl) hydrazine, trifluoroacetic acid salt (Life Technologies, Foster City, CA) in 22.4 mM phosphate buffer] were added to the samples and incubated at 50°C for 60 min in a thermal cycler. After purification, 750 ng biotin-labeled samples were used per Illumina HumanRef-8 chip array v3 (24,526 genes, 30 beads/chip). The hybridization cocktail was prepared according to the Bead Chip manufacturer’s instructions with the exception of the hybridization temperature, which was reduced to 48°C to accommodate the altered hybridization kinetics of cDNA/DNA pairs relative to cRNA/ DNA pairs.

#### Dasl WG

GEPs of RNA derived from FFPE samples were obtained using Illumina DASL WG v3.0 (24000 probes) (Illumina,). In brief, 200 ng of total RNA were converted to cDNA using biotinylated oligo-dT18 and random nonamer primers, followed by immobilization to a streptavidin-coated solid support. The biotinylated cDNAs were then simultaneously annealed to a set of assay-specific oligonucleotides. Extension and ligation of the annealed oligonucleotides generated PCR templates that were amplified using fluorescence-labeled and biotinylated universal primers. The labeled PCR products were then captured on streptavidin paramagnetic beads, washed, and denatured to yield single-stranded fluorescent molecules, which were hybridized to the WGgene expression BeadChips (HumanRef-8, Illumina) for 16 h at 58°C. The Illumina BeadArray Reader was used to scan the arrays, and Illumina BeadScan was used for image acquisition and recovery of primary data.

#### Affymetrix

Target preparation for Affymetrix GeneChip arrays was performed using 5 μg of amplified cDNA in 25 μL of nuclease-free water as input to the NuGEN Encore Biotin Module kit, according to the manufacturer’s recommendations.

Fragmented and labeled cDNA was hybridized onto Affymetrix HG-U133 Plus 2.0 gene chip, followed by washing and scanning.

A total of 5 μg in 50 μL of ssDNA labeled and fragmented with the NuGEN kit was used for each sample in the hybridization reaction. The hybridization cocktail was prepared according to the manufacturer’s instructions and was denaturated at 99°C for 5 min, then placed at 45°C for 5 min and finally spun at 14,000 rpm for 5 min before loading them on arrays. A total of 250 μL of the hybridization cocktail was loaded on arrays after a short GeneChip pre-hybridization in 1x hybridization buffer for 10 min in a rotating oven at 45°C and 60 rpm. Hybridization lasted 18 h in a rotating oven. GeneChips were scanned using the GeneChip Scanner 3000 (Affymetrix) using the manufacturer’s recommendations.

### Sample pre-assessment

To evaluate the quality of the amplified DNA obtained with WT-Ovation FFPE RNA Amplification System v2 (NuGEN) the expression of the *RPL13a* housekeeping gene was assessed using a TaqMan assay, which allows the amplification of a short sequence of 105 pb (Assay Id: Hs01926559_g1; Life Technologies). For each sample, 3.3 ng of amplified DNA were used in a 15-μl reaction volume, and the analysis was performed in triplicate. Real-time PCR was performed with the 7900HT Real-Time PCR System (Life Technologies) and the experiments were run using absolute quantification set-up. Data were analyzed by the SDS 2.4 software using a threshold of 0.2.

A small aliquot of DNA single strand product obtained with the WT-Ovation FFPE RNA Amplification System v2 was analyzed on the Agilent 2100 Bioanalyzer using the Agilent RNA 6000 NanoLabChip kit for an evaluation of amplicon size distribution. Only amplified DNAs with a significant amount of amplicons longer than 200 nt were used for the hybridization on Affymetrix; amplified DNAs with shorter amplicons were considered inadequate for further microarray analysis.

### Microarray data processing and statistical analysis

Data analysis was performed using R/Bioconductor software (version 2.13). Quality control on Illumina data and normalization, with the robust spline normalization method, were performed using the *lumi* package. For Affymetrix data, the Mas5 algorithm was applied and present calls computed using the *affy* package. All microarray data comply with MIAME guidelines and have been submitted to GEO (GSE38554).

Principal component analysis and hierarchical clustering (Euclidean distance and average linkage) were applied to evaluate similarity between samples. *ESR1* and *ERBB2* probesets ("205225_at" and "216836_s_at" respectively) were selected in accord with our previous reports [37,38] to evaluate ER and HER2 status.

In the analysis of the 44-sample cohort, only probesets detected in at least 10% of samples were retained. Genomic Grade Index was computed using the published gene list [39]. To identify breast cancer molecular subtypes, one probeset per gene (the one with the highest average value) was selected, and all PAM50 detected genes [40] were included in the hierarchical clustering analysis (Euclidean distance and average linkage).

### Building of an ER classifier

The supervised ER classification was obtained using the Prediction Analysis of Microarray (PAM) algorithm, a nearest shrunken centroid method [41]. Briefly, the classifier was trained on a dataset of 283 FF breast cancer samples. ER labels of this dataset were defined according to the expression of *ESR1* probeset 205225_at (threshold = 10.5). All *ESR1* probesets were filtered out before training the classifier. The optimal classifier was chosen according to accuracy evaluated by 10-fold cross-validation. The obtained classifier was applied to a public gene expression dataset of 127 FF breast cancer samples (GSE5460) and to our 44 FFPE-derived samples. The performance of the PAM ER classifier was evaluated in terms of accuracy, sensitivity, specificity and Cohen’s k, a measure of concordance, by comparing predicted ER status with immunohistochemical classification.

## Results

The study included three consecutive steps schematically summarized in S1 Fig, which: i) explored the possible use of two different Illumina gene expression platforms comparing GEPs obtained on FF samples with matched FFPE samples; ii) compared the use of Affymetrix HG-U133 Plus 2.0 chips with the DASL WG platform on FFPE samples, and iii) validated the feasibility of using FFPE samples in the clinical context in a pilot study including 60 samples.

### Explorative study to assess feasibility of measuring gene expression on FFPE samples with array platforms

Since FF samples represent the gold standard in array methods, our initial effort focused on comparison of GEPs in 10 matched frozen and FFPE samples. Frozen samples checked for RNA quality (RIN>7.5) were processed with the Illumina platform (HT-12; 48,803 probes) and compared to FFPE paired samples processed with the standard Illumina Ref-8 assay, which allows assessment of 24,526 probes (all included in the HT-12) with an increased bead/probe ratio compared to the HT-12 assay. Gene expression signals derived from FFPE samples are well known to be noisier than those of FF samples. Consequently, a higher gene/bead ratio, which warrants a higher number of replicate measures for each probe, could increase the reliability and precision of signals.

Taking into account the fact that the standard Illumina Ref-8 platform is not specifically designed for low-RNA-input samples, a linear amplification of RNA was performed using the WT-Ovation FFPE RNA Amplification System v2 kit prior to sample labeling and hybridization.

**Fig 1:**
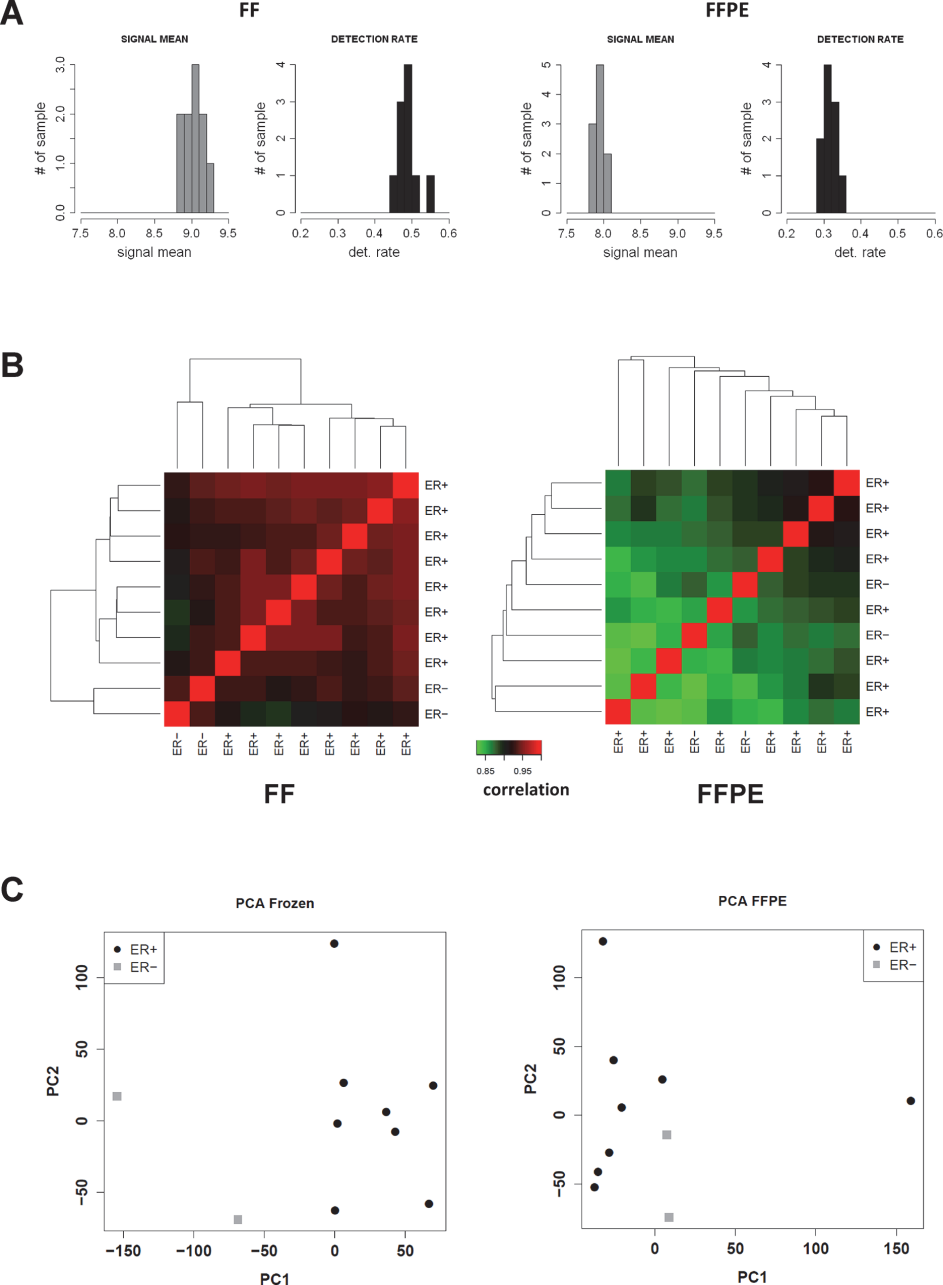
Signal intensities, reciprocal correlations and PCA plots for GEPs from FFPE and FF samples. A. Distribution plot of signal mean intensities (grey bars) and individual detection rates (black bars) in 10 FF samples profiled with the Illumina HT-12 chips and in 10 matched FFPE samples profiled with the Illumina Ref-8 chips. Only common probes were used. B. Heat map reporting the reciprocal correlations (Pearson correlation coefficient) among GEPs obtained from 10 FF breast cancer samples using Illumina HT-12 chips (left panel) and 10 matched FFPE samples profiled with Illumina Ref-8 chips (right panel). Prior to computation of the correlations, data were separately normalized using the robust spline normalization method C. PCA plots for GEPs obtained from 10 FF breast cancer samples with the Illumina HT-12 chips (left panel) and 10 matched FFPE samples profiled with the Illumina Ref-8 chips (right panel). Red circles represent ER+ samples and bleu circles represent ER- samples. Only samples with concordant ER status by RLA and IHC were included.

Data were analyzed considering only the 24,526 common probes between the HT-12 chips used for FF samples and the Ref-8 chips used for the FFPE samples. In FF samples the probe detection rate ranged from 0.45 to 0.55, whereas it hardly reached 0.35 for FFPE samples processed on the Ref-8 platform. Similarly, there was also a marked difference in the distribution of mean signals, with signal intensities derived from FFPE samples far below those obtained in FF samples (Fig 1A). Reciprocal correlations between samples are reported in Fig 1B for FF and FFPE samples. On the average, GEPs obtained from distinct individual FF samples were highly reciprocally correlated (Pearson’s r>0.9), and notably, the two ER-negative samples clustered separately. Individual gene profiles obtained for single FFPE samples using the Ref-8 chips were instead less strongly correlated (Pearson’s r<0.95), probably as a consequence of the lower quality of gene expression signals (lower mean signals and lower detection rate), which caused a higher noise level. No clustering according to ER status was observed.

By principal component analysis (PCA), FF sample-derived gene profiles sorted out the tumors according to ER status just by considering the first principal component, whereas for FFPE samples, a linear separation of samples by ER status was still possible, but with one mistake. Accordingly, the separation obtained with FF samples was rated as better. (Fig 1C).

Overall, such results led to the conclusion that, despite the introduction of linear amplification with the NuGEN kit specifically designed for FFPE samples, and despite the increased bead/probe ratio of the Ref-8 chips, the standard Illumina chips perform poorly on FFPE samples.

### Comparison between the DASL WG platform and the Affymetrix HG-U133 Plus 2.0 for measuring gene expression in FFPE samples

A second experiment was therefore run using 12 breast cancer samples (with only FFPE blocks available) aimed at comparing technical performance and biological reliability of the FFPE-dedicated Illumina WG DASL platform with that of Affymetrix chips (HG-U133 Plus 2.0). Six ER+ and 6 ER- cases (with concordant ER status by IHC and RLA) were selected, allowing an evaluation of the two different technical approaches based on their ability to identify known biological differences. The experimental design is reported in S1 Fig under step 2.

RNA was subjected to probe-specific amplification within the DASL assay and to linear amplification using the NuGEN WT-Ovation FFPE RNA Amplification System v2 kit prior to hybridization on the Affymetrix chips.

**Fig 2:**
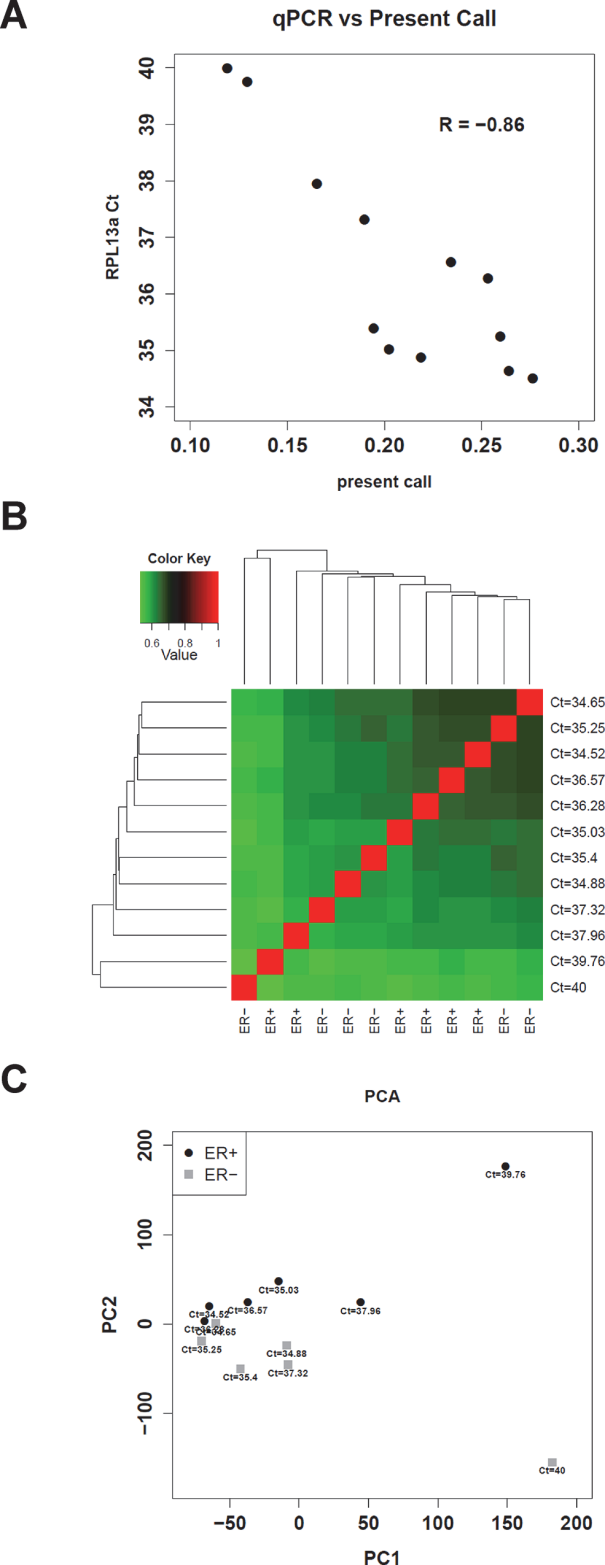
Diagnostic plots for GEPs obtained with Affymetrix arrays from 12 FFPE samples. **A.** RPLC13a qPCR threshold values (determined on the cDNA fraction prior to linear amplification) as a function of present call later obtained on Affymetrix HG-U133 2.0 Plus chips for 12 FFPE breast cancer samples. **B.** Heat map reporting the reciprocal correlations (Pearson correlation coefficient) between GEPs derived from 12 FFPE breast cancer samples using the Affymetrix HG-U133 2.0 Plus chips after MAS5 normalization. For each sample, ER status and Ct values referring to the sample-pre-assessment step with RPLC13 qPCR are also reported. **C.** PCA analysis plots of GEPS of 12 FFPE breast cancer samples profiled using the Affymetrix HG-U133 2.0 Plus chips. Black circles represent ER+ samples and grey squares represent ER- samples. Only samples with concordant ER status by RLA and IHC were included. RPL13a pPCR threshold values (Ct) are also reported.

According to recommendations from the manufacturer (ref: NuGEN Tecnical Report 1 http://www.nugeninc.com/nugen/index.cfm/linkservid/D94F0EDE-1C32-4916-A85B44333E35355F/), a sample pre-assessment step was done by running a qPCR for a house-keeping gene on cDNA. Based on preliminary results, the *RPL13a* gene was chosen for this purpose. Since we were in the process of defining optimal sample pre-assessment conditions for our set of 12 samples, all samples were hybridized irrespectively from Ct values obtained in the sample pre-assessment step. A linear correlation between qPCR-derived Ct values for the *RPL13* gene and Affymetrix present calls was obtained, which made it possible to derive a Ct cut-off value (Ct = 38) to pre-analytically assess the likelihood of obtaining samples with a present call value of at least 15%. Data are reported in Fig 2A. A second crucial step in pre-analytical quality assessment was run to evaluate the size distribution of DNA fragments obtained after RNA amplification, as there was a clear link between the size of amplified DNA samples and the present calls obtained after hybridization on the Affymetrix chips, which suggested that the size of amplified DNA was predictive of hybridization success. When the bulk of the amplified DNA fragments were short (< 200 nt), present call values dropped rapidly (data not shown).

Reciprocal correlations between all the 12 GEPs obtained with Affymetrix chips and PCA analysis are reported in Fig 2 (B and C). Ct values derived from the sample pre-assessment step reflect the quality of the profiles, and samples with the highest Ct values are the ones least correlated with the remaining samples. In PCA (Fig 2C) a separation according to ER status was however caught by the second component.

**Fig 3:**
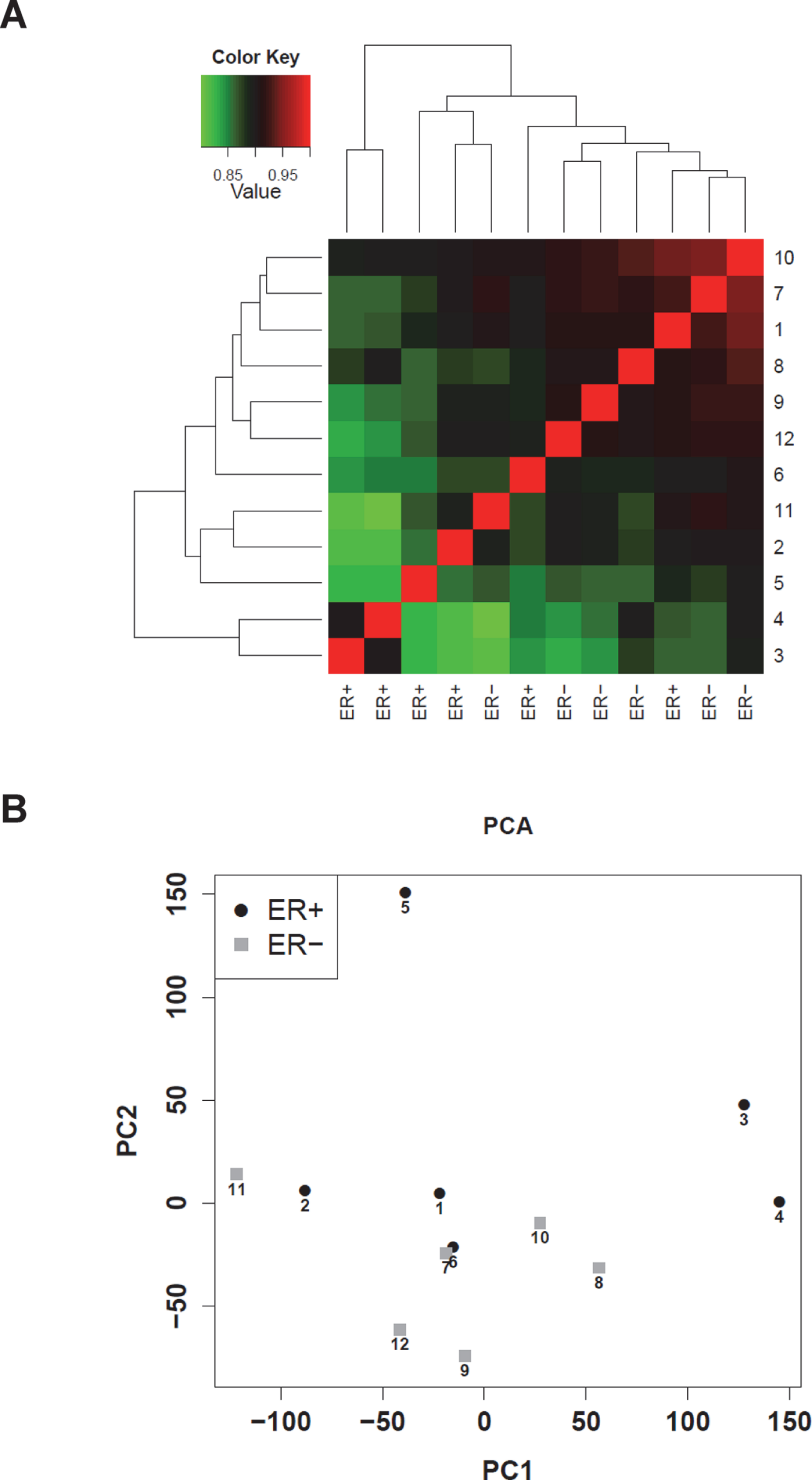
Reciprocal correlation and PCA plots for GEPs obtained with DASL WG from 12 FFPE samples. **A.** Heat map reporting the reciprocal correlations (Pearson correlation coefficient) between GEPs derived from 12 FFPE breast cancer samples using the Illumina DASL WG platform. For each sample ER status and sample ID (to allow comparability with panel B) were also reported. **B.** PCA plots of GEPS of 12 FFPE breast cancer samples profiled using the Illumina DASL WG platform. Black circles represent ER+ samples and grey squares represent ER- samples. Only samples with concordant ER status by RLA and IHC were included. Sample ID is reported.

RNA aliquots derived from the same samples were in parallel processed with the DASL platform. This time, detection rates were high and fairly similar in all samples (median 65%, range 60–70%). Reciprocal correlations and PCA (Fig 3) suggested some separations in GEPS, which did not however reflect ER status. Furthermore, there were two samples whose GEPs were poorly correlated with the remaining samples, but contrary to what expected, they did not coincide with those samples rated as poor quality when processed with Affymetrix chips.

**Fig 4:**
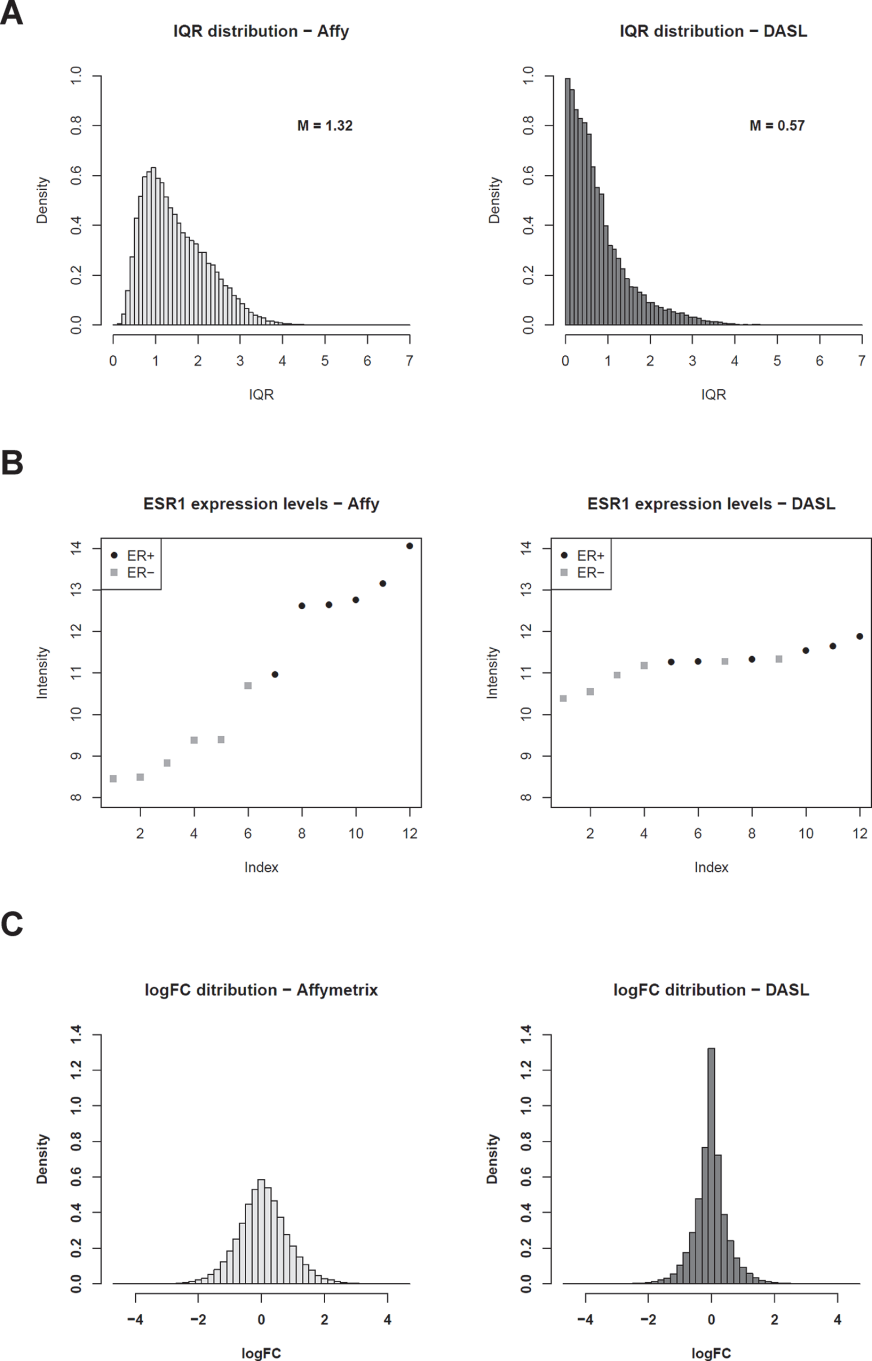
Comparison of reliability of GEPs obtained with Affymetrix and DASL WG platforms in FFPE samples. **A.** Distribution plots of interquartile ranges for GEPs obtained from 12 FFPE breast cancer samples using the Affymetrix HG-U133 2.0 Plus chips (left panel) or the Illumina DASL WG platform (right panel). **B.** Log_2_ expression intensity levels for ESR1in 12 breast cancer FFPE samples obtained respectively using Affymetrix HG-U133 2.0 Plus chips (left panel, ESR1 probeset ID “205225_at”) or the Illumina DASL WG platform (right panel, ESR1 probe ID “3360095”), from the lowest to the highest. Black circles and grey squares dot represent respectively ER+ and ER- negative samples, as determined by IHC. **C.** Distribution plots of log_2_-trasnformed fold change values obtained by Class Comparison between 6 ER+ and 6 ER- FF breast cancer samples profiled with Affymetrix HG-U133 2.0 Plus chips (left panel) or with the Illumina DASL WG platform (right panel).

GEPs obtained from DASL showed a lower variability in probe signals (median IQR = 0.57 vs median IQR = 1.32, *P*<2.2E-18, Wilcoxon test, Fig 4A) which was also confirmed looking at the *ESR1* gene alone (Fig 4B) with DASL-derived *ESR1* levels (defined using the address ID “3360095”) compressed in a narrower intensity interval compared to Affymetrix *ESR1* data and with a poor distinction (prediction) of ER classes (Fig 4B). Fold changes for all genes according to ER status were definitely higher in the Affymetrix data set, where a higher number of genes presented a fold change >2 or <0.5 (Fig 4C).

Based on the reported data, GEPs in the next samples were determined using the Affymetrix platform.

### Feasibility to profile FFPE samples collected within clinical trials with the developed technical protocol

Feasibility to profile FFPE samples derived from clinical trials was assessed on 60 samples from 10 to 14 year-old samples derived from primary breast tumors with known clinico-pathological information.

Five samples (8.3%) were lost after pathological assessment because they were judged as not representative of the tumor. The RNA extracted from the remaining 55 samples was not assessed for quality by the conventional Bioanalyzer electropherograms because high degradation and low RIN values were expected in all samples due to fixation. A careful quality control was instead run on the obtained cDNAs by evaluating by qPCR the RPL13A housekeeping gene to give an indication of chemical modifications which could impair RNA retro-transcription as already described.

Based on such combined criteria, an additional 7 samples were removed from the analysis and a total of 48 samples were hybridized on the chip. Two of the samples were characterized by ‘abnormal’ intensity levels, as shown from the box plots and clustered separately in a correlation plot of all samples, suggesting that their GEPs were unreliable (S3 Fig), and two samples were lost by the Service Provider during hybridization. The remaining 44 GEPs passed the quality control and showed median present call of 30%, ranging from 15 to 40%.

The validity of the developed quality control was confirmed here by the high hybridization success rate. Furthermore, after removal of the samples rated as poor quality, an inverse correlation (Pearson’s r = -0.62, N = 44) between Ct values for RPL13A and present call percentages on the Affymetrix chips (S2 Fig) was still observed.

**Fig 5:**
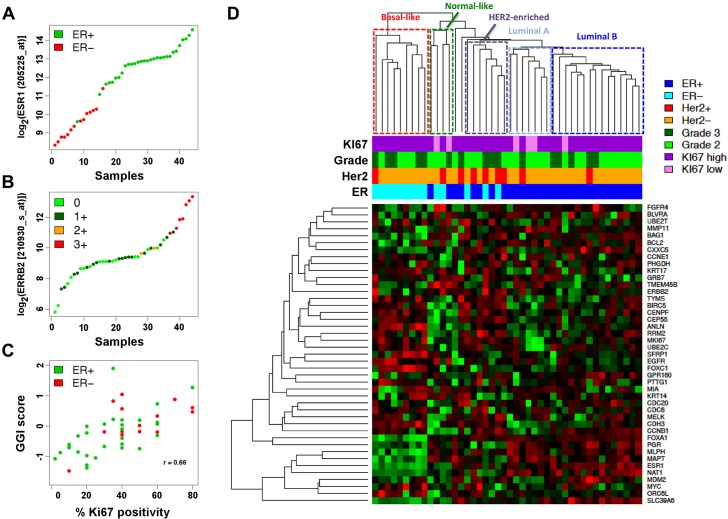
Biological reliability of Affmetrix FFPE data. A. Agreement between *ESR1* gene expression and IHC-derived ER status in the cohort of 44 good quality FFPE samples. B. Agreement between *ERBB2* gene expression and IHC-derived Her2 positivity in the same cohort. C. Comparison between the Genomic Grade Index and IHC-derived Ki67 positivity as independent estimators of tumor proliferation rate in the same cohort. D. Hierarchical clustering using the PAM50 gene signature. Samples belonging to the 5 molecular subtypes are indicated by dashed rectangles. Pathological definition of ER, Her2, Ki67 and grade are shown according to the color code on the right.

After quality control, we evaluated the biological reliability of the GEPs obtained from FFPE samples using several approaches. First, we correlated gene expression data with IHC characterization. *ESR1* gene expression was strictly correlated with IHC determined ER status (Fig 5A) and similarly, *ERBB2* gene values were in agreement with IHC positivity (Fig 5B). Tumor proliferation rate was estimated on gene expression data by computing the Genomic Grade Index [39] and compared with pathologically assessed Ki67 positivity; a strong positive correlation (R = 0.66, Spearman correlation) was found (Fig 5C).

We also tested whether FFPE-derived gene expression data were able to identify breast cancer molecular subtypes. To this aim, we performed a hierarchical cluster analysis including genes from the PAM50 signature [40] detected in our dataset. Despite the relatively small sample size and the absence of G1 tumors in our cohort, we were still able to identify clusters of patients belonging to the 5 molecular subtypes (Fig 5D).

One of the most common uses of array profiling in clinical studies is the classification of samples based on transcriptional signatures. To evaluate such capability in our FFPE-derived GEPs, we developed a gene expression-based ER status classifier by applying the PAM algorithm to GEPs of 193 ER+ and 90 ER- FF samples obtained from the expO breast cancer dataset (GSE2109). *ESR1* probesets were excluded from the training phase and the final classifier, which included 36 probesets, showed a 10-fold cross-validation accuracy of 95.8%. The predictive accuracy of this ER classifier was then assessed in an independent cohort of 127 FF breast cancer samples (GSE5460), by comparing the predicted ER status with the immunohistochemical classification. Results showed that the classifier was able to assign the ER status of patients with a high accuracy (Table 1). The same classification was performed on our FFPE-derived cohort and we still observed high performances in separating ER+ and ER- patients (Table 1).

**Table 1:** Performance of the ER classifier.

Predicted ER status	GSE5460		FFPE pilot study	
	ER*-	ER*+	ER*-	ER*+
**ER-**	50	1	14	4
**ER+**	3	73	0	26
**Accuracy**	96.85		90.91	
**Sensitivity**	0.94		0.87	
**Specificity**	0.99		1.00	
**Cohen’s k**	0.94		0.81	

*ER status by immunohistochemistry

## Discussion

Numerous solutions are now available for obtaining GEPs in FFPE samples, ranging from specifically designed arrays such as DASL assay (Illumina) and Almac Xcel Array (Affymetrix) to alternative approaches based on RNA-seq [42]. The latter is a promising and rapidly developing approach for transcriptome studies, but data analysis is still poorly standardized and time consuming compared with microarrays, a critical point when dealing with the profiling of large cohorts with translational purposes. Furthermore, the use of an array platform offers the unquestionable advantage of comparing FFPE data with previous clinical datasets. In particular, the widely used Affymetrix chips were originally designed for good quality RNA, but several literature data [16,18,19,43,44] support their use also with degraded samples, with some modifications of the protocol preceding hybridization.

Independently of the chosen solution, before starting gene expression studies on such types of biological samples, a careful study plan including direct assessment of the chosen method and a pilot study to evaluate in advance failure rates and biological relevance of obtained data, are necessary. Good technical solutions for profiling FFPE samples alone are indeed not sufficient to warrant success when applying this type of approach to clinical studies as, by definition, FFPE sample are noisier sources of material. Indeed, age of samples, the time-length of fixation and of course the time elapsed between surgical removal and fixation, are all major sources of variability, which represent to a certain extent a study-specific challenge.

Prediction of single sample failure is an additional issue and, although the general failure rate can be foreseen by running pilot studies on the same type of samples, failure of individual samples is optimally controlled only by sample pre-assessment procedures. Again, sample pre-assessment strategies cannot be blindly derived from other similar studies, and although pre-assessment is strictly dependent on the methods employed, its value should be better confirmed in each specific study.

In keeping with the aforementioned considerations, we have reported here pre-study considerations for planning a GEP study for an optimal exploitation of information obtainable from FFPE samples collected within clinical trials. The developed protocol herein described has been successfully applied to the European Cooperative Operable Breast Cancer trial [45].

The first comparison was run between GEPs obtained from FFPE data and those obtained from matched FF samples using the Illumina platform routinely available in our Functional Genomics Unit. Processing with a platform designed for good quality RNA did not result in the expected separation according to ER status, despite the use of the Ref-8 platform characterized by a lower number of probes but a higher bead:probe ratio, and low mean signals and detection rates were obtained. Consequently, information derived from GEPs obtained with the Ref-8 chips was valued as unsatisfactory.

We intentionally did not look in more depth at the GEPs obtained from FFPE and FF samples as such type of data have already been reported in various contexts [18,19,43,46]. Instead, we focused on the independent type of information obtainable from each type of sample.

We therefore continued our technical assessment experiments using FFPE samples only and comparing a well-established assay specifically developed for degraded RNA samples [24,47] with the Affymetrix chips, thereby exploiting a pre-amplification strategy specifically designed for FFPE samples [16].

Actually, both the DASL assay and our protocol included an amplification step, which is potentially introducing biases in the results. However, the amplification bias is minor when using linear amplification as with the NuGEN approach compared to the exponential amplification of the DASL protocol. The choice of the reverse transcription approach plays a major role in FFPE samples. Since using oligo(dt) primers alone to target the 3’end of mRNA molecules may not be optimal due to the addition of mono- methylol groups to the adenosine residues during formalin fixation, we preferred to use the NuGEN approach, where a mixture of poly(dT) and random primers is used. This was also guided by the specific design of the array platform, where all probes mapped within 600 bp from 3'-end. With this approach, constraints due to chemical modification of the poly(A) tail are overcome and amplification of rRNA is limited.

Interestingly, although the Affymetrix approach has not been designed for degraded RNA, we demonstrated that under controlled conditions it can give very meaningful results. This was mainly shown by its better correlation with IHC/RLA-determined ER expression data and larger fold changes compared to DASL, a consequence of the narrower dynamic range of the latter. A note of caution should however be raised due to the small number of samples used for the direct comparison between DASL and Affymetrix approaches. Accordingly, our conclusions on the comparison between the two approaches should be regarded as context specific, and our preference for the Affymetrix platform derives from a combination of many criteria. We are aware that the DASL approach gave important contributions in the clinical arena [25–31], but at the same time it should be mentioned that while we were preparing the manuscript Illumina interrupted production of the DASL assay.

Based on such preliminary comparative studies, the main focus of our effort was to run a pilot study with the more promising platform (of the two investigated) to evaluate the real possibility to profile retrospectively samples collected within clinical trials. The pilot study was therefore run with 60 samples completely comparable to those to be used in future clinical studies. This allowed us to calculate the failure rate due to sample histological representativeness, RNA or GEP quality. Globally, 27% of our samples were lost to translational purposes (5 were judged as non-representative by the pathologist, 7 did not pass the pre-assessment step, 2 did not reach quality standards after hybridization on the array, and 2 were simply lost by the Service Provider during processing), but sample pre-assessment avoided unnecessary hybridizations of 7 samples (13% of samples rated with good histological quality). At this point, 96% of samples passed the post-hybridization quality control, testifying to the utility of the pre-assessment strategy developed within our sample processing workflow.

Importantly, we were able to demonstrate the biological reliability of the 44 profiles rated as good quality samples. Indeed, we found excellent correlation of *ESR1* and *ERBB2* and proliferation gene expression data with independent IHC characterization. In keeping with this, unsupervised clustering using the PAM50 signature [40] led to the identification of samples belonging to the 5 molecular subtypes that showed the characteristic expression patterns. As stated before, performing FFPE-derived expression profiles using the same platform previously employed to profile large cohorts of FF breast cancer samples gives the possibility to develop clinically relevant predictors in the latter, which can be validated in FFPE cohorts, possibly collected within clinical trials, thereby speeding up the biomarker development process [34]. To demonstrate the feasibility of such an approach, we generated and validated in GEPs from frozen samples an ER status classifier that was successfully tested also in our FFPE cohort.

## Conclusions

Taken together, our results fully support the use of the Affymetrix approach in FFPE samples derived from clinical trials and demonstrate that these types of samples correctly processed, submitted to pre-assessment steps and possibly optimally analyzed [48] can give reliable and biologically meaningful information ready to be used for clinical purposes, without a too high loss in the number of samples. This represents thus a valid approach to be used in clinical studies also in the discovery phase and to identify specific signatures to be later transferred to simpler technical approaches such as IHC or Nanostring.

However, the facts that FFPE data are noisier and that a percentage of samples can be lost at several steps support the need to plan studies including a higher number of samples with respect to those predicted to be necessary to attain the predefined statistical power. The pre-assessment protocol applied in the present study represents a valid tool to prescreen clinical samples in order to identify those not likely to give reliable GEPs. This may limit the increase in costs due to the necessity to increase the sample size.

## Supporting Information

S1 Fig Outline of the study experimental design and set of samples used. (TIF)

S2 Fig Correlation between Ct values and present calls. RPLC13a qPCR threshold values (determined on the cDNA fraction prior to linear amplification) as a function of present calls later obtained on Affymetrix HG-U133 2.0 Plus chips for 44 FFPE breast cancer samples from our pilot study.
(TIF)

S3 Fig Quality control on raw data for GEPs derived from 46 FFPE breast cancer samples. GEPs from the pilot study using Affymetrix HG-U133 2.0 Plus chips are reported. A. Box plot of raw data intensities. B. Heat map reporting the reciprocal correlations (Pearson correlation coefficient).
(TIF)
