# Supplementary figures and images for: Use of Formalin-Fixed Paraffin-Embedded Samples for Gene Expression Studies in Breast Cancer Patients
Source: PLoS One. 2015 Apr 6;10(4):e0123194. doi: 10.1371/journal.pone.0123194 (PMC4386823; doi:10.1371/journal.pone.0123194)

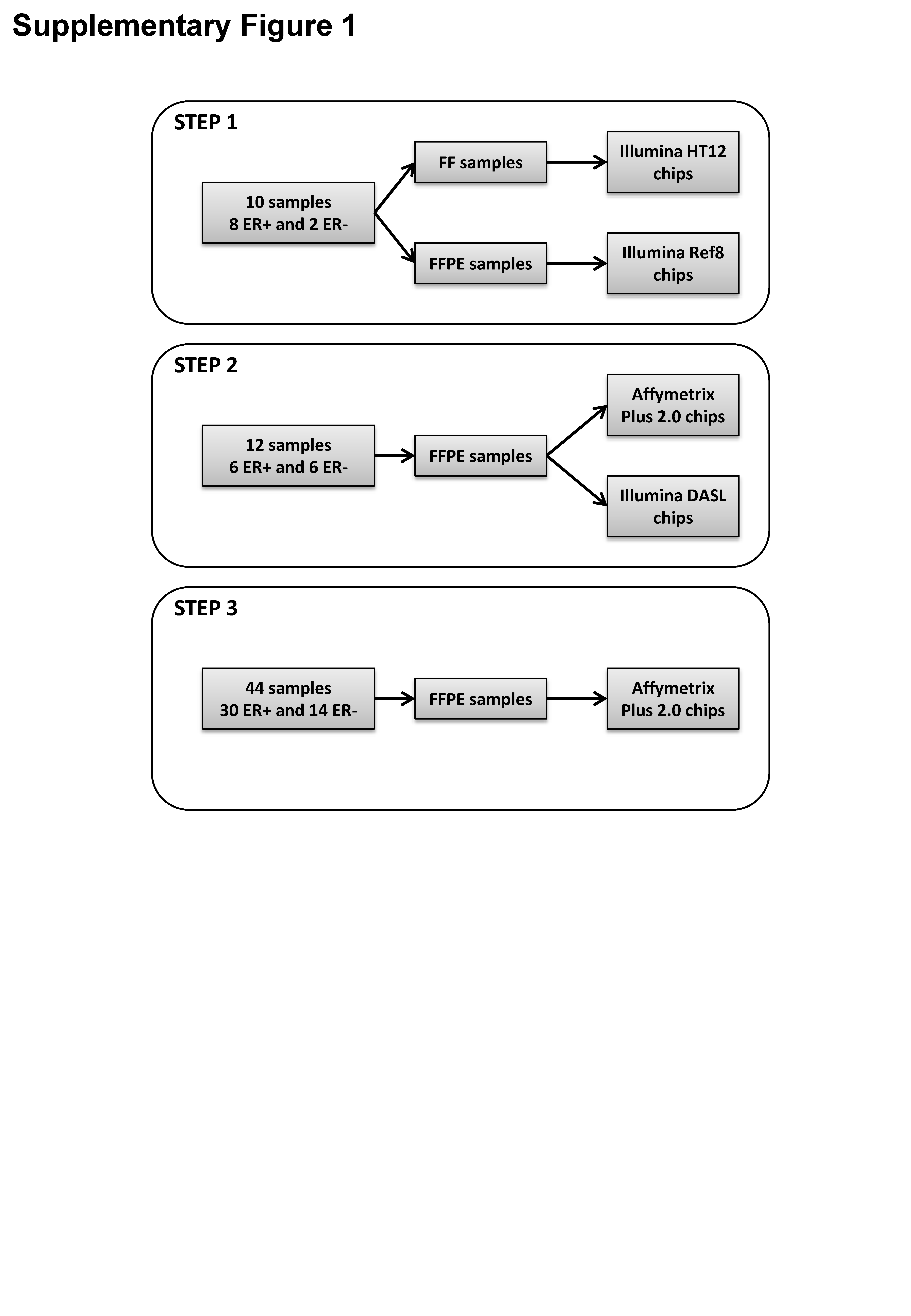

Supplement: S1 Fig — (TIF) [file pone.0123194.s001.tif]

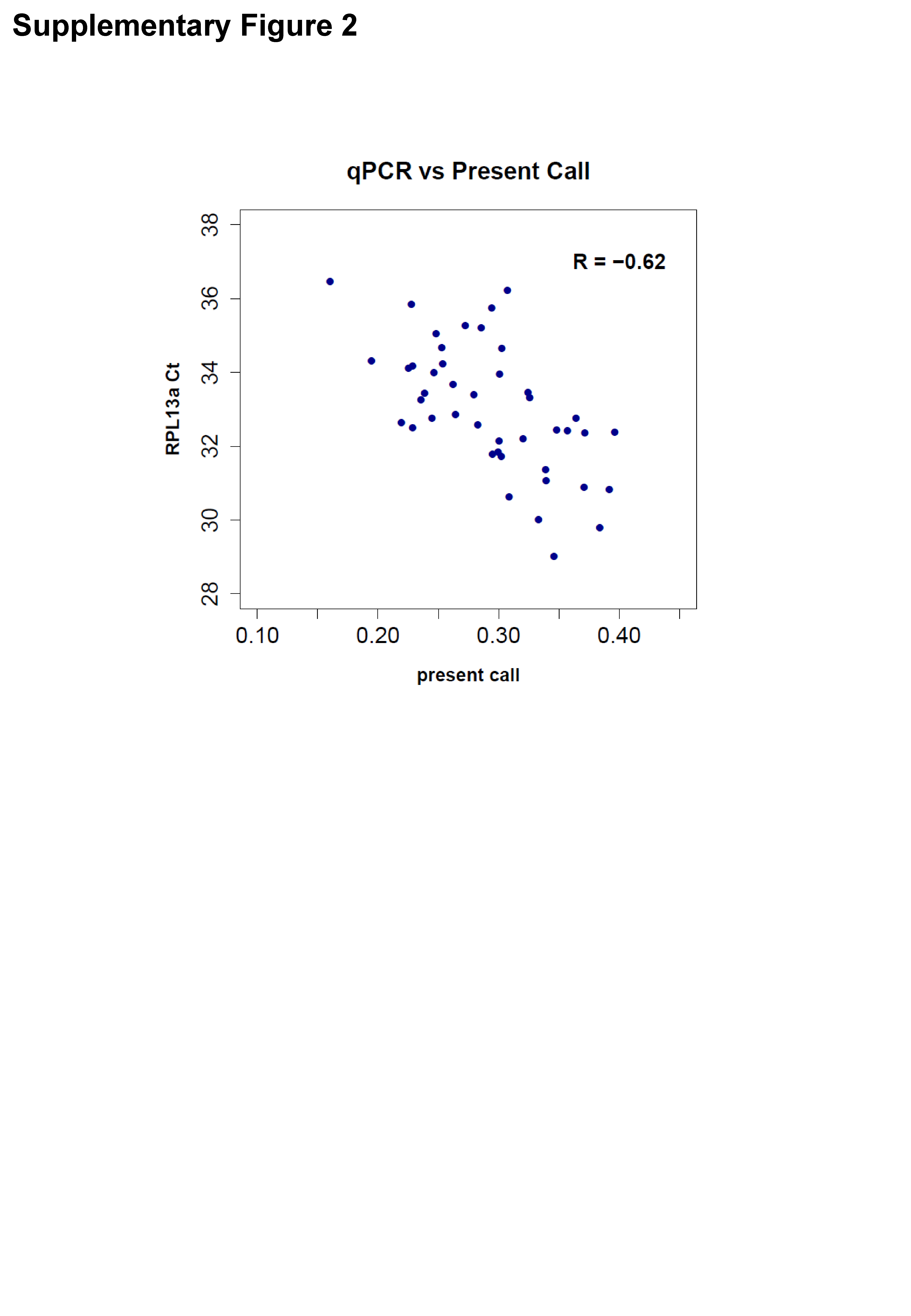

Supplement: S2 Fig — RPLC13a qPCR threshold values (determined on the cDNA fraction prior to linear amplification) as a function of present calls later obtained on Affymetrix HG-U133 2.0 Plus chips for 44 FFPE breast cancer samples from our pilot study. (TIF) [file pone.0123194.s002.tif]

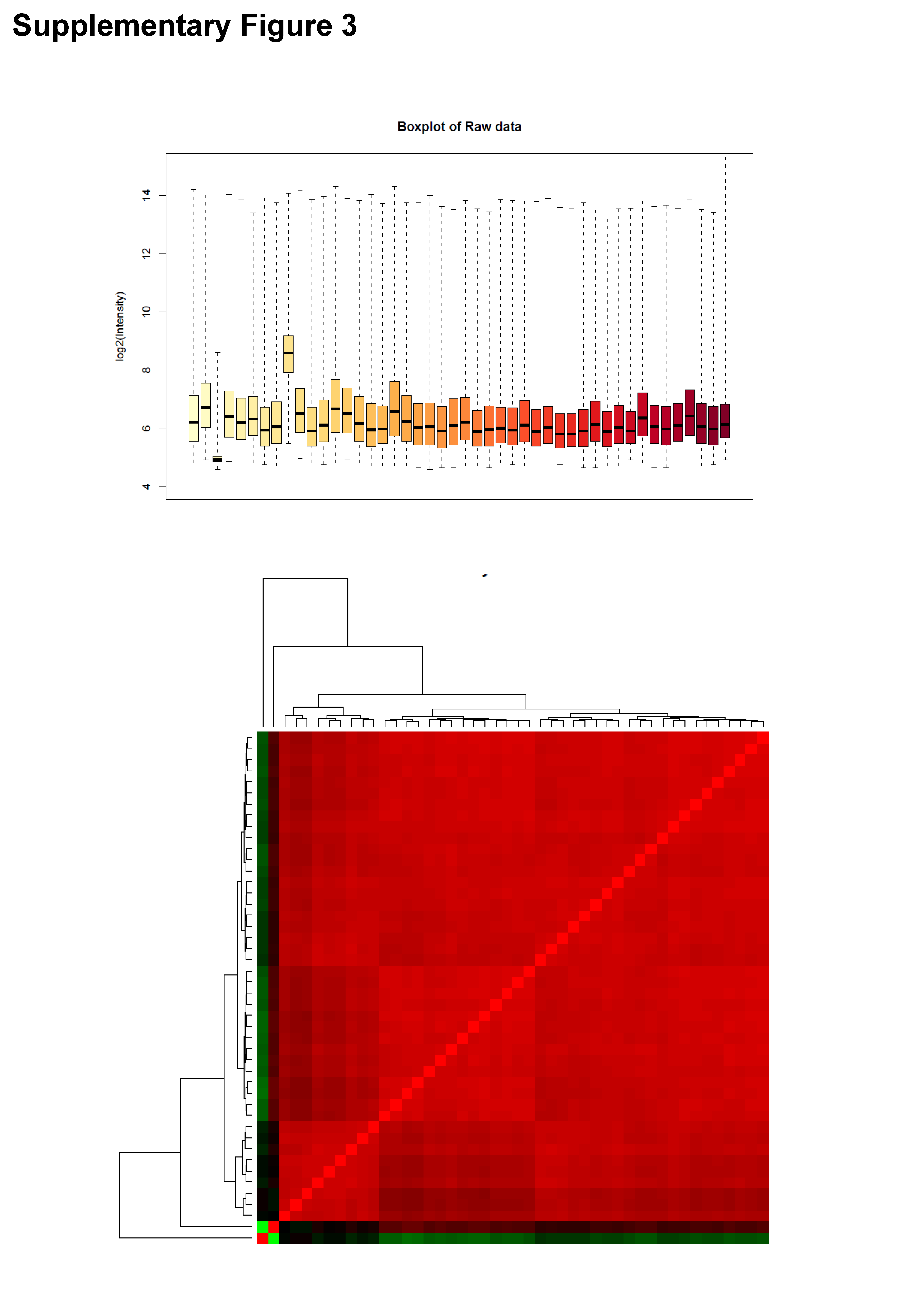

Supplement: S3 Fig — GEPs from the pilot study using Affymetrix HG-U133 2.0 Plus chips are reported. A. Box plot of raw data intensities. B. Heat map reporting the reciprocal correlations (Pearson correlation coefficient). (TIF) [file pone.0123194.s003.tif]
